# Anti-TACI single and dual-targeting CAR T cells overcome BCMA antigen loss in multiple myeloma

**DOI:** 10.1038/s41467-023-43416-7

**Published:** 2023-11-18

**Authors:** Rebecca C. Larson, Michael C. Kann, Charlotte Graham, Christopher W. Mount, Ana P. Castano, Won-Ho Lee, Amanda A. Bouffard, Hana N. Takei, Antonio J. Almazan, Irene Scarfó, Trisha R. Berger, Andrea Schmidts, Matthew J. Frigault, Kathleen M. E. Gallagher, Marcela V. Maus

**Affiliations:** 1 Cellular Immunotherapy Program, Massachusetts General Hospital, Boston, MA USA; 2 Harvard Medical School, Boston, MA USA; 3 Cancer Center, Massachusetts General Hospital, Boston, MA USA; 4 Department of Pathology and Center for Cancer Research, Massachusetts General Hospital and Harvard Medical School, Boston, MA USA

**Keywords:** Immunotherapy, Cancer, Myeloma

## Abstract

Chimeric Antigen Receptor (CAR) T cells directed to B cell maturation antigen (BCMA) mediate profound responses in patients with multiple myeloma, but most patients do not achieve long-term complete remissions. In addition, recent evidence suggests that high-affinity binding to BCMA can result in on-target, off-tumor activity in the basal ganglia and can lead to fatal Parkinsonian-like disease. Here we develop CAR T cells against multiple myeloma using a binder to targeting transmembrane activator and CAML interactor (TACI) in mono and dual-specific formats with anti-BCMA. These CARs have robust, antigen-specific activity in vitro and in vivo. We also show that TACI RNA expression is limited in the basal ganglia, which may circumvent some of the toxicities recently reported with BCMA CARs. Thus, single-targeting TACI CARs may have a safer toxicity profile, whereas dual-specific BCMA-TACI CAR T cells have potential to avoid the antigen escape that can occur with single-antigen targeting.

## Abstract (web summary)

Patients with myeloma multiple treated with BCMA CAR T cells often relapse with BCMA-negative disease or antigen escape. Here the authors describe the design of TACI-directed single and dual CAR T cells with in vitro and in vivo activity against multiple myeloma, overcoming BCMA antigen loss.

## Introduction

Multiple myeloma is defined as a plasma cell disorder and accounts for more than 10% of all hematologic malignancies^1^. Despite improvements in available therapies with high-dose chemotherapy and autologous stem cell transplant, until recently, multiple myeloma was considered largely incurable and characterized by recurring relapses of increasingly refractory disease^2–6^.

CAR T cell therapy has emerged as a revolutionary therapeutic option for multiple myeloma, with two recent FDA approvals^7,8^ targeting B cell maturation antigen (BCMA), a member of the TNF receptor superfamily^9^. However, this therapy is not curative either, in line with other single-targeted therapies^10–13^. Recent evidence suggests that some patients treated with BCMA CAR T cells relapse with BCMA-negative disease, or antigen escape^14–17^. In addition, there is evidence that BCMA is strongly downregulated and trogocytosed after exposure to BCMA CAR T cell therapies, suggesting that targeting of a second antigen may be warranted^18^.

In the setting of CD19-directed CAR T cells, multiple approaches to overcoming CD19-negative relapse have been explored, including CARs targeting two antigens, such as CD19 and CD20^19^, CD19 and CD22^20^, or CD19 and CD79b^21^. Based on the promising results with BCMA targeting in multiple myeloma, we explored targeting TACI, a related member of the TNF receptor superfamily that provides plasma cells with survival signals^22^. TACI is found at high levels on most myeloma cells^23^ and is predominantly expressed on plasma cells. We and others^24^ have previously devised an approach to target BCMA and TACI with CAR T cells using its natural ligand, A Proliferation-Inducing Ligand (APRIL), which binds both BCMA and TACI. A CAR construct using a single moiety of APRIL as the binding domain of the CAR, however, did not show efficacy in a clinical trial (NCT03287804^25^). The recent publication of the results of this study indicated that although APRIL protein had reasonable binding affinity for BCMA that was comparable to a single-chain variable fragment, APRIL-based CARs induced poor T cell activation, weak cytokine production, and had reduced binding avidity to tumor cells. Thus, although TACI has significant potential as a target for multiple myeloma, the design of the APRIL CAR was suboptimal^26^. We developed a trimeric form of APRIL, termed TriPRIL, which more closely mimics the trimer state of the natural ligand^23^, and is currently in clinical development (NCT05020444^27^). One potential concern with using APRIL-based CARs is that an ineffective cytotoxicity event following synapse formation between the T cell and the tumor cell could result in the amplification of a survival signal to the tumor cell based on signaling through BCMA and/or TACI; however, our pre-clinical data and others^18^ did not find any evidence of this hypothetical concern.

The first scFv-based CAR T cell targeting BCMA (idecabtagene vicleucel, or ide-cel) achieved impressive clinical results but is generally not considered curative^10^. More recently, a bi-paratopic, single-domain antibody approach (ciltacabtagene autoleucel, or cilta-cel) has led to even deeper and more durable responses. However, recent reports describe an unexpectedly high incidence (≥5%) of a severe Parkinsonian-like toxicity after treatment with FDA-approved BCMA CARs^28,29^. Subsequent correlative studies from autopsy samples of a patient who succumbed to this toxicity demonstrated low but detectable expression of BCMA in the basal ganglia^30^. This expression was validated in a cohort of healthy donor tissue as well. Based on a brain atlas dataset, we confirmed that TACI appears to be a safe target without significant expression in the basal ganglia, where we could also confirm detectable BCMA expression.

Here we describe the development of a second-generation CAR T cell against the multiple myeloma target TACI. We immunize mice with TACI protein, test resulting hybridoma antibodies for specificity to TACI-expressing cells, and use the antibody sequence to design scFvs in CAR format. Anti-TACI CAR T cells are cytotoxic in vitro and in vivo in xenograft models of multiple myeloma. We show that TACI expression is retained on multiple myeloma cells when BCMA expression is lost in both knockout cell lines and in a patient with relapsed multiple myeloma plasmacytoma after treatment with anti-BCMA CAR, further supporting the use of TACI as an alternative CAR T cell target in BCMA-negative malignancies. To overcome or prevent relapse due to antigen loss, we design dual-specific tandem scFv CAR T cells targeting both BCMA and TACI. We show that these dual-targeting CAR T cells are efficacious against wildtype multiple myeloma cell line models. Even in the context of antigen loss, dual-targeting CAR T cells can retain anti-tumor activity in cell lines and in a plasma cell tumor from a patient who relapsed after BCMA-targeted CAR T cell therapy. Thus, targeting two antigens may provide a more effective long-term strategy for CAR T cell therapy for multiple myeloma, without increasing the risk of Parkinsonian-like neurotoxicity.

## Results

### Evaluating TACI expression in the brain

**Fig. 1:**
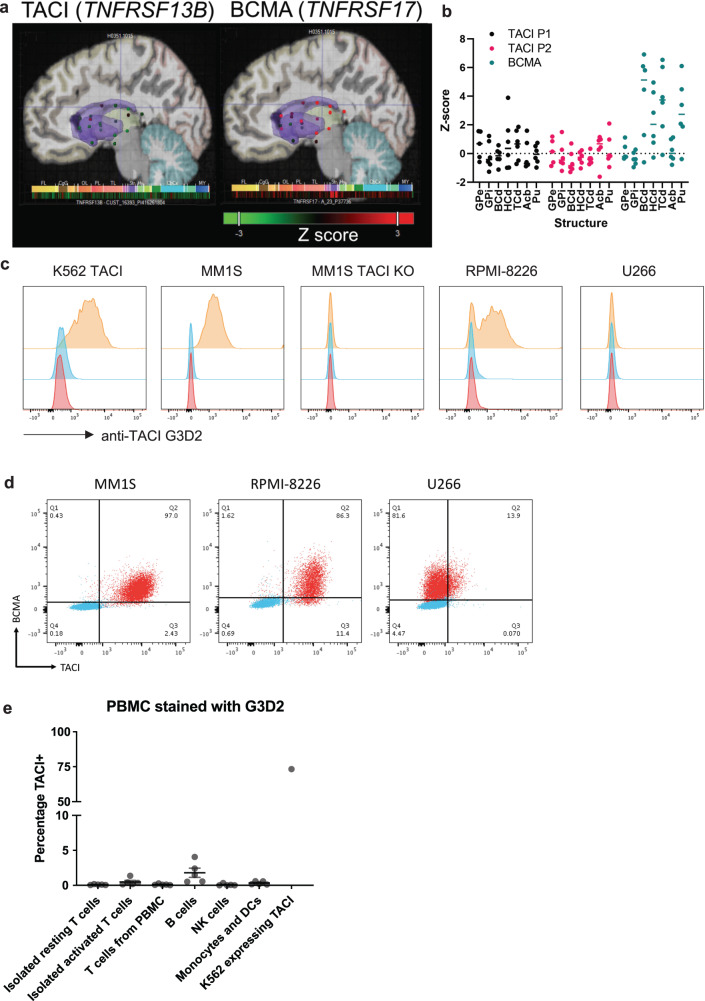
TACI expression in the basal ganglia is limited and the anti-TACI antibody G3D2 specifically stains multiple myeloma. **a** Spatial representation of RNA expression in the basal ganglia (indicated in purple) from one representative donor (H0351.1015). Colormap indicates Z-score (Allen Human Brain Atlas Brain Explorer 2). Structure abbreviations: FL frontal lobe, CgG cingulate gyrus, OL occipital lobe, PL parietal lobe; TL temporal lobe, Str striatum, Hy hypothalamus, CbCx cerebellar cortex, MY myelencephalon. **b** RNA expression across basal ganglia structures. Data from two distinct probes for TACI - P1 and P2 - were available. Each point for the indicated structure indicates one human donor (*n* = 6). Expression Z-scores were queried from the Allen Brain Atlas Data Portal. Structure abbreviations: GPe globus pallidus external segment, GPi globus pallidus internal segment, BCd body of caudate nucleus, HCd head of caudate nucleus, TCd tail of caudate nucleus, Acb nucleus accumbens, Pu putamen. **c** G3D2 staining of cell lines transduced with membrane-bound TACI protein and TACI-expressing multiple myeloma cell lines (anti-TACI with secondary in orange, isotype with secondary in blue, secondary alone in red). **d** BCMA and TACI staining of K562-TACI and multiple myeloma cell lines using commercially available conjugated flow antibodies (stained cells in red, isotype stain in blue). **e** G3D2 staining of vital immune cells present in PBMC or activated T cells (*n* = 5ND, +/– SEM). Raw data is provided in the Source Data file. ND = normal donor.

Single cell analysis from the Human Protein Atlas^31,32^ showed enriched expression *TNFRSF13B*, the gene that codes for TACI, in plasma cells and B cells (Supplementary Fig. 1), consistent with prior reports and known expression patterns^23^. One principle of cancer-targeted therapies is to avoid overlapping toxicity profiles. Due to the recent reports of BCMA-associated movement and neurocognitive toxicities^30^, we profiled TACI expression at the protein and RNA levels. Protein datasets did not show expression of either BCMA or TACI^33,34^. However, we did observe BCMA RNA expression in the basal ganglia using the Allen Human Brain Atlas^35^ microarray data at much higher levels than TACI, which was negligible (Fig. 1a, b). In the six normal donors profiled, we found consistent and high expression of BCMA in the body, head, and tail of the caudate nucleus as well as the putamen. The negligible expression of TACI relative to BCMA across the caudate nucleus and putamen suggests reduced risk of on-target, off-tumor toxicity in the basal ganglia with this target.

### Anti-TACI antibody development

We generated an antibody targeting TACI by immunizing immunocompetent mice with recombinant TACI protein and sequencing the resulting hybridoma, clone G3D2. Using surface plasmon resonance, the *K*_D_ of G3D2 binding to TACI was determined to be 8.61 × 10−^10^ M (Supplementary Fig. 2). We tested the binding of the G3D2 soluble antibody by staining K562 cells transduced to overexpress TACI as well as a panel of human multiple myeloma cell lines (MM1S, RPMI-8226, and U266) and evaluated binding by flow cytometry (Fig. 1c). To confirm the specificity of binding, we used CRISPR/Cas9 engineering to knock out TACI from MM1S cells (TACI KO). The G3D2 antibody proved to be specific in its cell surface staining of TACI, with positive stains for high-level TACI expression (K562 TACI) as well as endogenous-level expression (MM1S, RPMI-8226). G3D2 showed no staining when TACI was knocked out (MM1S TACI KO). One multiple myeloma cell line, U266, had no detectable TACI expression with the G3D2 antibody. We also stained the multiple myeloma lines with commercially available antibodies targeting TACI, which showed expression on K562-TACI, MM1S, and RPMI-8226 and low expression on U266 (Fig. 1d). BCMA was expressed on all multiple myeloma cell lines. We did not detect expression of either BCMA or TACI on K562 WT cells (Supplementary Fig. 3a).

Expression of TACI on subsets of B cells and T cells has been reported^36,37^. To determine if G3D2 could potentially target other immune cells, we stained peripheral blood mononuclear cells (PBMCs) from five normal donors and evaluated binding to resting and activated T cells, B cells, NK cells, monocytes, and dendritic cells, and compared the percentage of TACI+ cells to K562s overexpressing TACI (Fig. 1e). Of these cell types, we only observed minor staining of CD19^+^ B cells (less than 2%). We also did not observe staining of TACI on CAR T cells which are activated during production (Supplementary Fig. 3b).

### Anti-TACI CAR T cells are efficacious in vitro and in vivo against wildtype multiple myeloma

**Fig. 2:**
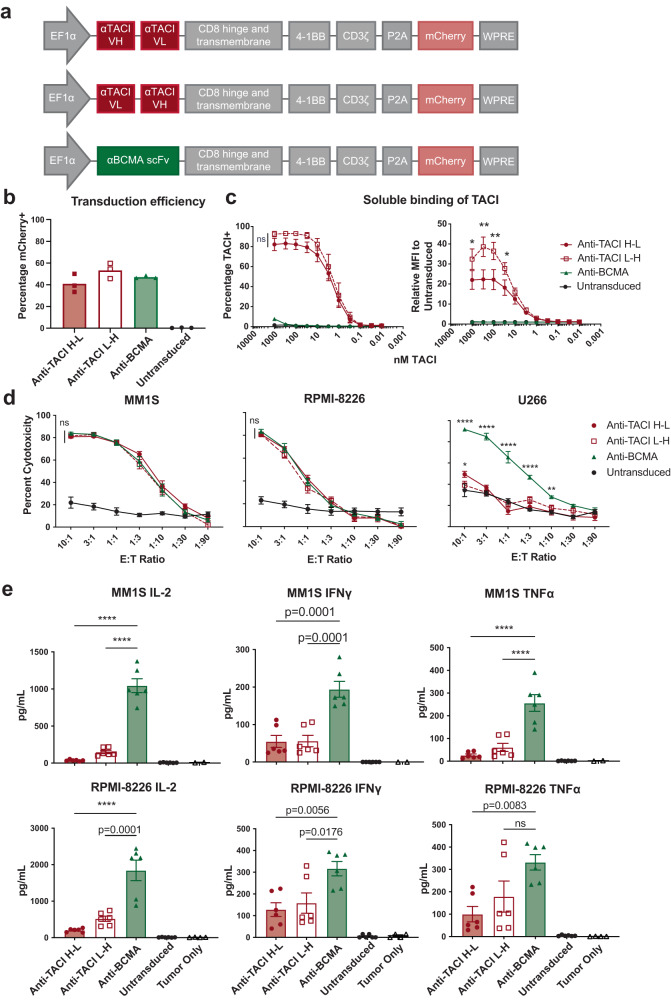
Anti-TACI CARs are functional against TACI+ cell lines in vitro with lower cytokine production than anti-BCMA CARs. **a** Anti-TACI and control anti-BCMA CAR T cell design. **b** Transduction efficiency measured by flow cytometry reported by mcherry of human normal donor T cells transduced with anti-TACI or anti-BCMA CAR T cells (*n* = 3ND). **c** Binding of soluble TACI to anti-BCMA and anti-TACI CARs at the indicated concentrations measured by flow cytometry. Percentage of TACI+ mcherry+ CAR T cells shown on left and MFI of FITC on mCherry+ CAR T cells on right (*n* = 3ND+/– SEM, paired t test at each concentration; ns at all concentrations between H-L and L-H anti-TACI CAR percentage on left, *=0.047921 at 1000 nM, **=0.002989 at 300 nM, **=0.009396 at 100 nM, and *=0.029103 at 30 nM between H-L and L-H anti-TACI CAR MFI on the right). **d** Luciferase-based cytotoxicity assays of multiple myeloma cell lines targeted by anti-TACI or BCMA CAR T cells at the indicated E:T ratios after 18 h of coculture (*n* = 3ND in biological triplicate +/– SEM, unpaired t tests at each ratio, ns between anti-TACI and anti-BCMA CARs for MM1S and RPMI-8226 at all E:T ratios; lowest significance shown for U266 where *=0.044010 and **=0.002825). Calculated as a percentage of luminescence of tumor only wells. **e** IL-2, IFNγ, and TNFα cytokine production from supernatant of 1:1 E:T coculture for 18 h (*n* = 3ND in biological duplicate +/– SEM, one way ANOVA compared to control anti-BCMA CAR). Raw data is provided in the Source Data file. E:T = effector:target cell; ns = non-significant. All tests are two-sided. *p* = ****<0.0001.

Based on sequencing the variable chains of the G3D2 antibody, we designed CAR T cells targeting TACI. We created two orientations of the heavy (H) and light (L) variable chains (Fig. 2a) linked with 4 repeats of G4S. Throughout experiments, we compared the anti-TACI CARs to tool anti-BCMA CAR constructs we made based on the patent literature describing bb2121/ide-cel. Across multiple normal donors, anti-TACI CARs had similar transduction efficiencies to that of anti-BCMA CAR (Fig. 2b). We tested the ability of the CARs to bind soluble TACI protein and observed increased binding of the L-H anti-TACI CARs compared to the H-L version (Fig. 2c). Soluble TACI did not bind anti-BCMA CAR or untransduced T cells (UTD) from the same normal donors. We tested the anti-TACI CAR T cells against these cell lines in vitro and observed cytotoxicity against MM1S and RPMI-8226 but much lower activity against U266, consistent with the lack of binding of our antibody to U266 (Fig. 2d). This suggests these CAR T cells are specific to targeting TACI on multiple myeloma cell lines. Interestingly, across both MM1S and RPMI-8226, the anti-TACI CARs had lower cytokine production of IL-2, IFNγ and TNFα compared to anti-BCMA CARs (Fig. 2e).

**Fig. 3:**
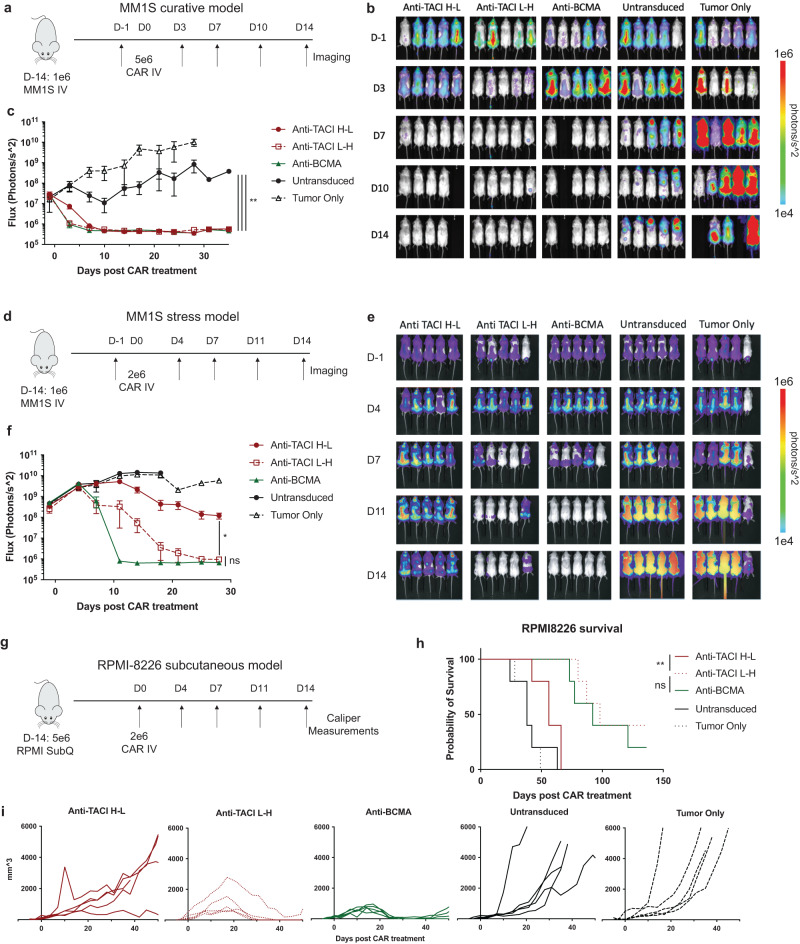
Anti-TACI L-H CAR shows superior efficacy in vivo against stress models of multiple myeloma. **a** Schematic of MM1S curative model treated with 5e6 CAR+ cells intravenously 14 days after intravenous engraftment of 1e6 MM1S cells. **b** BLI imaging of a. **c**, Quantified BLI of a (5 mice per group +/– SEM, unpaired t test to Untransduced at day 35 where **=0.004935 for anti-TACI H-L, **=0.001696 for anti-TACI L-H, **=0.004928 for anti-BCMA). **d** Schematic of MM1S stress model treated with 2e6 CAR+ cells. **e** BLI imaging of **d**. **f** Quantified BLI of d (5 mice per group +/– SEM, experiment representative of 3 separate experiments each treated with a different ND, unpaired t test at day 28 between anti-TACI H-L and anti-TACI L-H where *=0.014174). **g** Schematic of RPMI-8226 subcutaneous model treated with 2e6 CAR+ cells intravenously 14 days after subcutaneous engraftment of 5e6 RPMI-8226 cells. **h** Survival of g (log-rank Mantel-Cox test **=0.0026). **i** Caliper measurements of individual mice from **g** for the first 50 days (5 mice per group, experiment representative of 2 separate experiments each treated with a different ND). Raw data is provided in the Source Data file. All tests were two-sided unless otherwise stated.

We next tested the anti-TACI CARs in a xenograft model of multiple myeloma using luciferized MM1S, which homes to the bone marrow when injected intravenously (IV). Two weeks after MM1S cell injection, we injected CAR T cells IV and imaged animals biweekly to assess tumor burden. Anti-TACI CAR T cells were comparable to anti-BCMA and were able to clear animals of tumor (Fig. 3a–c). To elucidate differences between the anti-TACI CAR orientations, we tested these two versions in a stress model of MM1S by using a lower dose of CAR T cells. Here we observed that the L-H version had more potent anti-tumor activity (Fig. 3d–f). While anti-BCMA CARs had faster clearance of tumor in this model compared to anti-TACI L-H, by day 28 there was no significant difference in tumor flux.

We expanded our findings to a third xenograft model using subcutaneous engraftment of the RPMI-8226 multiple myeloma cell line. Using caliper measurements to monitor tumor growth over time, we again observed that L-H anti-TACI CARs were superior at controlling tumor growth and promoting survival compared to H-L CARs, and were comparable to anti-BMCA CARs (Fig. 3g–i).

### BCMA-negative multiple myeloma can be targeted by anti-TACI CAR T cells

**Fig. 4:**
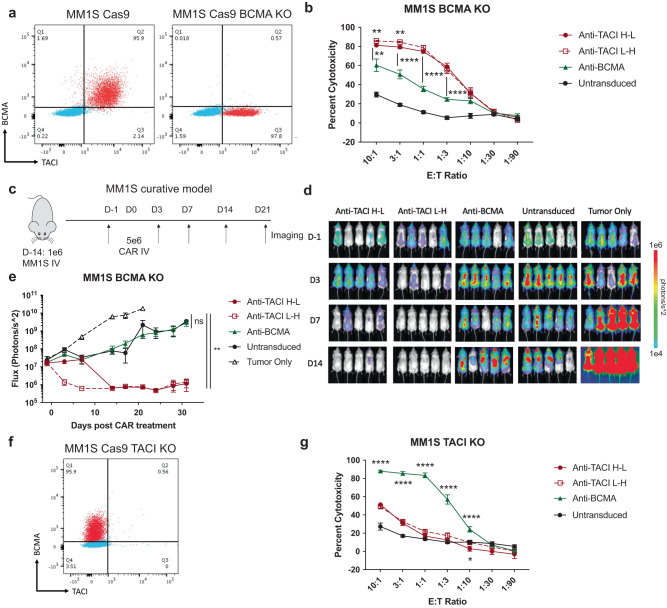
Anti-TACI CARs are functional against BCMA negative multiple myeloma but lose efficacy against TACI negative tumor. **a** BCMA and TACI flow cytometry staining of MM1S Cas9+ WT and BCMA KO cells. **b** Luciferase-based cytotoxicity assay of MM1S BCMA KO cell line targeted by anti-TACI or BCMA CAR T cells at the indicated E:T ratios after 18 h of coculture (*n* = 3ND+/– SEM in biological triplicate, unpaired t tests at each ratio where **=0.005797 between anti-BCMA and anti-TACI H-L and 0.001331 between anti-BCMA and anti-TACI L-H and 0.001224 between anti-TACI H-L and anti-TACI L-H at a 10:1 E:T and **=0.002689 between anti-TACI H-L and anti-TACI L-H at a 3:1 E:T). Calculated as a percentage of luminescence of tumor only wells. **c** Schematic of MM1S BCMA KO curative model treated with 5e6 CAR+ cells. **d** BLI imaging of **c**. **e** Quantified BLI of c (5 mice per group +/– SEM, experiment representative of 3 separate experiments each treated with a different ND, unpaired t test to Untransduced at day 31 where **=0.002040 for anti-TACI H-L and **=0.005764 for anti-TACI L-H). **f** BCMA and TACI flow cytometry staining of TACI KO MM1S Cas9+ cells. **g** Luciferase-based cytotoxicity assay of MM1S Cas9+ TACI KO cell line targeted by anti-TACI or BCMA CAR T cells at the indicated E:T ratios after 18 h of coculture (*n* = 3ND+/– SEM in biological triplicate, unpaired t tests at each ratio where *=0.045779). Calculated as a percentage of luminescence of tumor only wells. Raw data is provided in the Source Data file. All tests are two-sided. *p* = ****<0.0001.

Since BCMA loss has been reported in the clinic after treatment with anti-BCMA CAR T cells^14–17^, we wanted to test if the anti-TACI CAR T cells were functional in the context of antigen loss. We generated MM1S BCMA KO tumor using CRISPR/Cas9 technology and observed that BCMA-negative cells retained TACI expression (Fig. 4a). In in vitro cytotoxicity assays, anti-BCMA CAR T cells had diminished cytotoxicity against the BCMA-negative line, but anti-TACI CAR T cells remained equally as cytotoxic (Fig. 4b). We next tested the efficacy of these CAR T cells in vivo and again observed that anti-TACI CAR T cells could clear the BCMA negative tumor while anti-BCMA CAR T cells were comparable to untransduced T cells (Fig. 4c–e). It is conceivable that targeting TACI alone could result in TACI loss, as has been observed with prior single-targeting CAR T cell therapies. We tested the anti-TACI CAR T cells against MM1S TACI KO cells in vitro and confirmed loss of cytotoxic activity (Fig. 4f, g). These data also suggest, but do not confirm, that TACI-CARs have lower tonic signaling than BCMA-CARs, which we confirmed using in vitro cytotoxicity assays against the T cell SupT1 line (Supplementary Fig. 4).

### Dual-specific CAR T cells directed against BCMA and TACI are efficacious when either antigen is lost

**Fig. 5:**
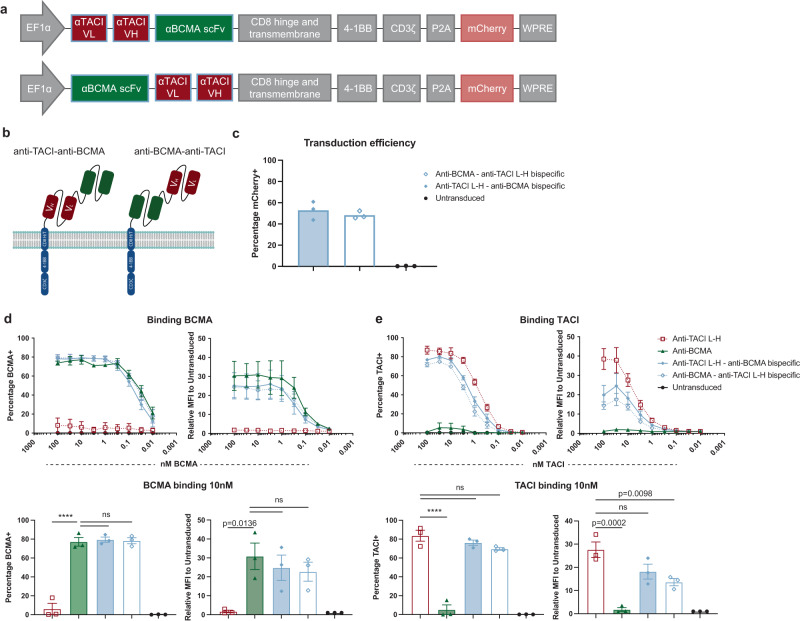
Bispecific CAR T cells have high transduction efficiency and high binding of soluble antigen. **a** Schematic of bispecific CAR T cell vector design. **b** Model of tandem scFv CAR T cells. **c** Transduction efficiency of dual-targeting CARs (*n* = 3ND). Binding of soluble BCMA (**d**) or TACI (**e**) to bispecific CARs at the indicated concentrations measured by flow cytometry (*n* = 3ND+/– SEM). Binding at 10 nM shown below for simplicity (one way ANOVA compared using anti-BCMA or anti-TACI L-H CARs as controls accordingly). Raw data is provided in the Source Data file. All tests are two-sided. *p* = ****<0.0001.

To mitigate potential relapse due to antigen loss with monospecific targeting, we decided to target both BCMA and TACI using tandem scFv bispecific CAR T cells containing the superior L-H version of the anti-TACI CAR. We created two bispecific CARs, changing the proximity of the anti-TACI and anti-BCMA scFvs to the transmembrane domain and linking the two scFvs with 4 repeats of the flexible G4S linker (Fig. 5a, b). Despite the larger vector size, the transduction efficiency of the dual-targeting CAR constructs was comparable to that of single scFvs in normal donor T cells (Fig. 5c). The tandem dual-specific scFv-based CARs had comparable binding to soluble BCMA as anti-BCMA CARs and soluble TACI to anti-TACI L-H CARs at the population level. The dual-specific CARs had slightly reduced binding of TACI by MFI compared to the mono-specific anti-TACI CAR (Fig. 5d, e).

**Fig. 6:**
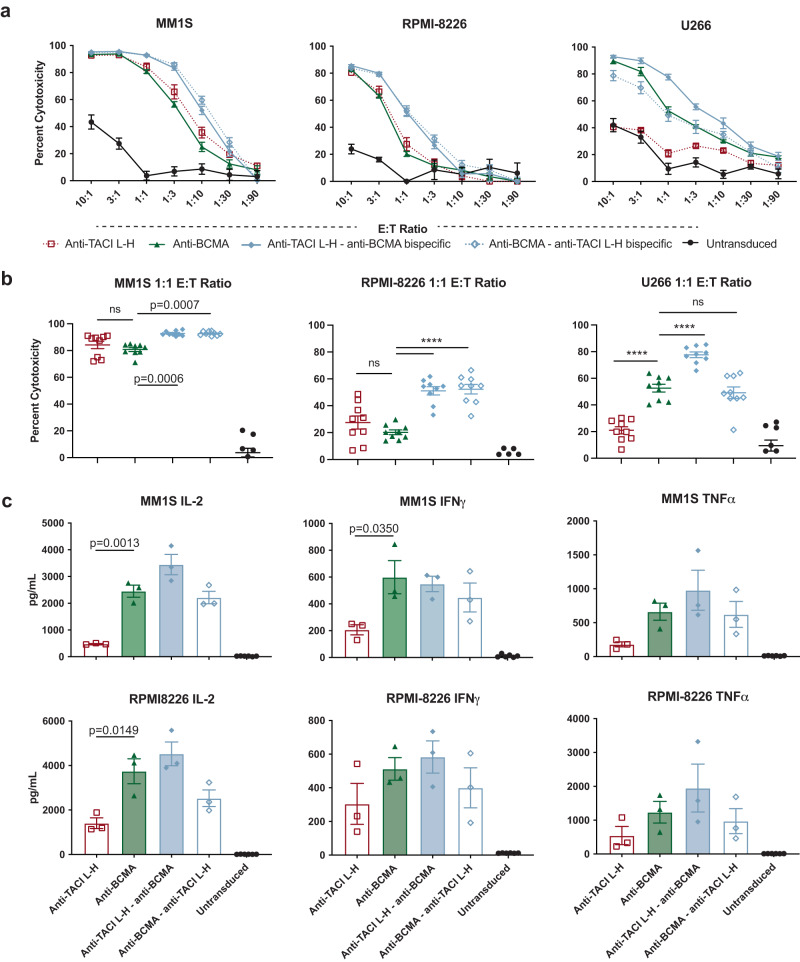
Bispecific CAR T cells targeting BCMA and TACI are efficacious against wildtype multiple myeloma in vitro. **a** Luciferase-based cytotoxicity assays of MM1S, RPMI-8226, and U266 cell lines targeted by CAR T cells at the indicated E:T ratios after 18 h of coculture (*n* = 3ND+/– SEM in biological triplicate). Calculated as a percentage of luminescence of tumor only wells. **b** Cytotoxicity from a at the 1:1 E:T ratio (*n* = 3ND+/– SEM in biological triplicate, one way ANOVA compared to control anti-BCMA CAR). **c** IL-2, IFNγ, and TNFα cytokine production from supernatant of 1:1 E:T cell coculture with the indicated CAR T cells and tumor cells for 18 h (*n* = 3ND+/– SEM, one way ANOVA compared to control anti-BCMA CAR, ns not shown). Raw data is provided in the Source Data file. All tests are two-sided. *p* = ****<0.0001.

Both of the tandem dual-specific CAR T cells had higher in vitro cytotoxicity compared to monospecific anti-BCMA and anti-TACI L-H CAR T cells against wildtype multiple myeloma cell lines MM1S and RPMI-8226 (Fig. 6a, b). Interestingly, only anti-TACI–anti-BCMA bispecific CAR T cells were more cytotoxic against TACI^low^ multiple myeloma line U266 compared to anti-BCMA CAR T cells. At the 1:1 effector:target (E:T) cell ratio, anti-BCMA–anti-TACI bispecific CAR T cells had equivalent killing to anti-BCMA CAR T cells. There were no significant differences in cytokine production observed between the anti-BCMA and dual-targeting CAR T cells (Fig. 6c).

**Fig. 7:**
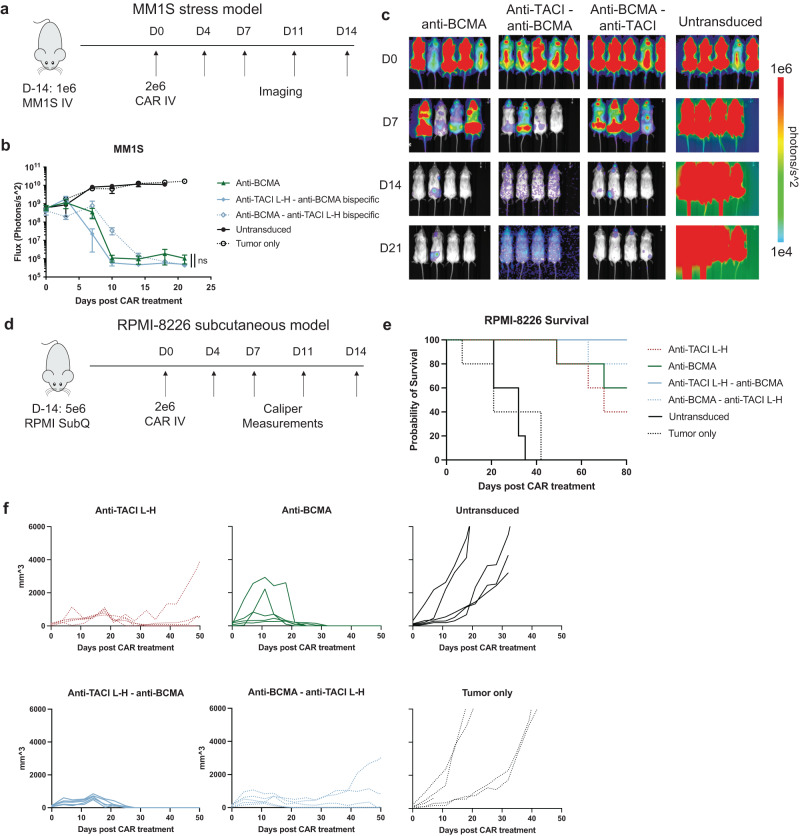
Bispecific CAR T cells are efficacious against wildtype multiple myeloma in vivo. **a** Schematic of MM1S stress model treated with 2e6 CAR+ cells. **b** Quantified BLI of a (5 mice per group +/- SEM, unpaired t test at day 18). **c** Images from **a**, **b**, **d** Schematic of RPMI-8226 subcutaneous model (5 mice per group). **e** Survival of **d**, **f** Caliper measurements from **d** Raw data is provided in the Source Data file. All tests are two-sided. These data are representative of two separate experiments.

We also tested the dual-targeting CAR T cells in two of the xenograft multiple myeloma models used earlier. In the stress model against MM1S, the tandem bispecific CAR T cells controlled tumor growth similar to anti-BCMA CAR T cells (Fig. 7a–c). The anti-TACI–anti-BCMA bispecific CAR T cells showed faster kinetics in reducing tumor burden (for example, at Day 10), though by Day 18 there were no significant differences among the treatment groups. In the subcutaneous RPMI model, the bispecific CAR T cells also delayed tumor growth and increased survival (Fig. 7d–f). Again, the anti-TACI–anti-BCMA CAR T cells showed slightly improved tumor control compared to the anti-BMCA–anti-TACI version. We also evaluated CAR T cells expanding in the blood of these animals at day 28 post treatment. We did not observe any significant differences in CAR T cell expansion, memory phenotype or exhaustion marker expression (Supplementary Fig. 5).

**Fig. 8:**
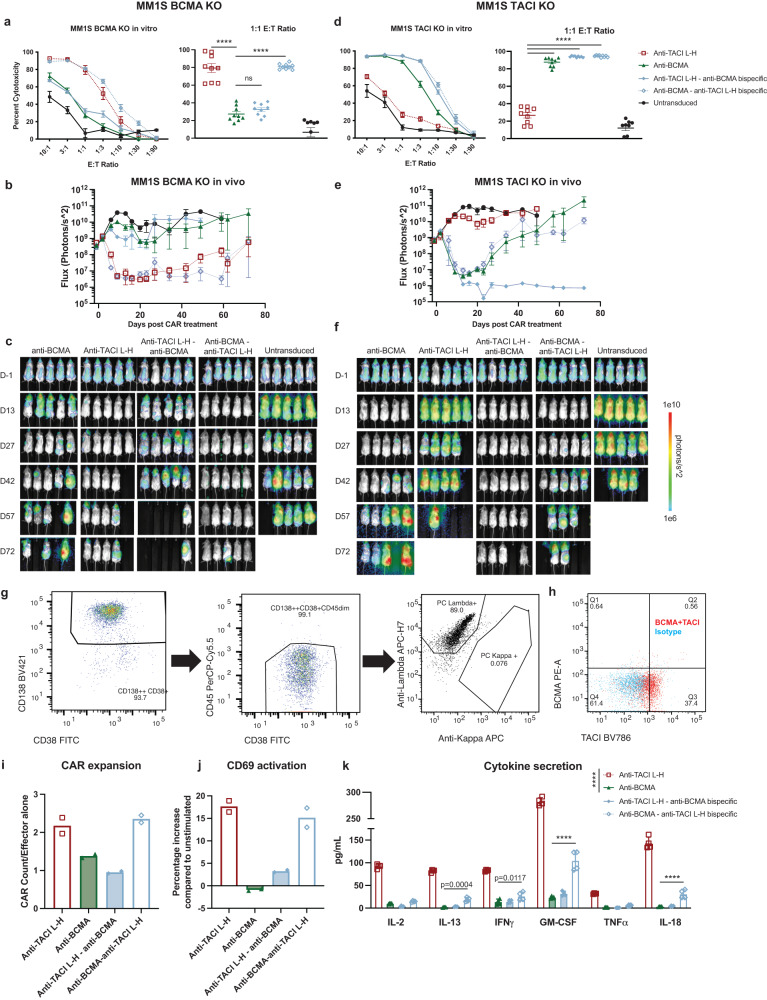
In the context of antigen loss, anti-BCMA – anti-TACI tandem bispecific CARs maintain efficacy. **a** Luciferase-based cytotoxicity assays of BCMA KO MM1S targeted by bispecific CAR T cells at the indicated E:T cell ratios after 18 h of coculture (*n* = 3ND+/– SEM in biological triplicate, one way ANOVA compared to control anti-BCMA CAR). Calculated as a percentage of luminescence of tumor only wells. **b** Quantification of BLI from MM1S stress model with BCMA KO tumor treated with bispecific CARs (5 mice per group +/– SEM). **c** Images of **b**. **d** Luciferase-based cytotoxicity assays of TACI KO MM1S targeted by bispecific CAR T cells at the indicated E:T cell ratios after 18 h of coculture (*n* = 3ND+/– SEM in biological triplicate, one way ANOVA compared to control anti-TACI CAR). Calculated as a percentage of luminescence of tumor only wells. **e** Quantification and images of BLI from MM1S stress model with TACI KO tumor treated with bispecific CARs (5 mice per group +/– SEM). **f** Images of **e**. The in vivo experiments shown are representative of two separate experiments. **g** Gating of relapsed patient multiple myeloma cells from an extramedullary biopsy of a lesion of increasing size after treatment with ciltacabtagene autoleucel. After forward and side scatter gating, cells were gated on CD138++ CD38+CD45dim and evaluated as lambda or kappa. **h** Patient myeloma cells from **g** stained for BCMA and TACI (BCMA + TACI stained cells in red, isotype in blue). Anti-TACI L-H, anti-BCMA and bispecific CARs were cocultured with patient myeloma cells from **g**, **h** at a 2:1 E:T ratio for 24 h and evaluated via flow cytometry for CAR expansion measured by mCherry (**i**) and activation with CD69 expression (**j**) (1ND in biological duplicate). **k** Cytokine analysis from supernatants of cocultures (1ND+/– SD in four biological replicates, two way ANOVA compared to control anti-BCMA CAR; anti-BCMA v anti-TACI L-H *p* = **** and anti-BCMA v anti-TACI-anti-BCMA *p* = ns for all). Raw data is provided in the Source Data file. All tests are two-sided. *p* = ****<0.0001.

Finally, we investigated how these bispecific CAR T cells behaved when BCMA or TACI antigen was lost. We utilized MM1S KO tumors and tested the CARs in vitro and in vivo. In these antigen-escape scenarios, loss of BCMA showed the greatest difference among the constructs, with the anti-TACI–anti-BCMA bispecific losing efficacy, indicating greater dependency on the presence of BCMA antigen for this CAR (Fig. 8a). However, the anti-BCMA–anti-TACI CAR retained the activity of single targeting anti-TACI CAR T cells against MM1S BCMA KO in vitro. In vivo, anti-BCMA–anti-TACI CARs were able to have extended control of MM1S BCMA KO tumor in the stress model whereas anti-TACI–anti-BCMA CARs were not (Fig. 8b, c). When TACI antigen was knocked out, both bispecific CARs were initially able to clear tumor in vitro and in vivo, but both anti-BCMA and anti-BCMA–anti-TACI CARs eventually relapsed while anti-TACI–anti-BCMA CARs mediated sustained tumor clearance (Fig. 8d–f). We hypothesize that the proximal scFv to signaling has a stronger effect on CAR function, and the initial response by both bispecific constructs suggests loss of TACI is not as impactful on dual-specific CAR activity.

There is some concern that targeting TACI will not have a strong effect due to the lack of clinical efficacy of APRIL-based CARs^25^ but we previously generated ligand-based, trimeric APRIL (TriPRIL) CARs to improve binding and function relative to monomeric APRIL-based CARs. Here we also compared TriPRIL CARs to our tandem scFv bispecific anti-BCMA–anti-TACI CAR. We observed lower binding of soluble BCMA to TriPRIL CARs compared to anti-BCMA–anti-TACI and remarkably lower binding of TACI as well (Supplementary Fig. 6a). In a subcutaneous model of RPMI-8226, we observed stronger efficacy and survival of animals treated with anti-BCMA–anti-TACI CAR compared to TriPRIL CAR (Supplementary Fig. 6b, c), suggesting the tandem scFv based bispecific could have higher potency than TriPRIL, though only clinical data could test this hypothesis.

Finally, we identified a patient treated with ciltacabtagene autoleucel, as per the standard of care, who relapsed after CAR treatment. Plasma cells were isolated from a core biopsy of an expanding extramedullary lesion and cryopreserved. We evaluated the tumor cells for BCMA and TACI expression and discovered the patient had relapsed with BCMA-negative disease but had significant expression of TACI (Fig. 8g, h). We cocultured the patient’s cells with anti-TACI based CARs to evaluate CAR response in terms of expansion, activation, and cytokine secretion. While anti-BCMA and anti-TACI–anti-BCMA CARs had limited activity against the tumor cells, anti-TACI and anti-BCMA–anti-TACI CARs had a significant increase in CAR expansion and activation (Fig. 8i, j). In addition, anti-TACI CARs had a significant cytokine response with increased levels of IL-2, IL-13, IFNγ, GM-CSF, TNFα, and IL-18 compared to anti-BCMA CARs. Anti-BCMA–anti-TACI CARs had a more modest response with a significant increase of IL-13, IFNγ, GM-CSF, and IL-18. This shows the superiority of the anti-BCMA–anti-TACI orientation for dual-specific design in the context of BCMA loss and the functionality of anti-TACI CARs in the setting of BCMA-negative relapsed disease.

## Discussion

BCMA-targeted CAR T cell therapy has been efficacious as a treatment for multiple myeloma, leading FDA approval of two BCMA-directed CAR T cell products^7,8^. Despite the overall success rate, reports of patients relapsing with BCMA-negative disease after BCMA-directed CAR T cell therapy are now emerging^14–17^. This constitutes a significant obstacle for BCMA-directed therapies. Efforts to target additional antigens are becoming increasingly important. Some groups have identified SLAMF7^38^ and GPRC5D^39^ as targets, which have been tested in clinical trials^40,41^ (NCT03958656^42^, NCT04555551^43^). Another antigen, TACI, has also been identified as a potentially favorable CAR target due to high expression on multiple myeloma, but has not yet been tested in the form of single-chain variable fragment CARs^44^. To avoid antigen escape, CARs targeting both BCMA and TACI simultaneously are in clinical development, but so far only using natural ligand (APRIL) of these molecules as the extracellular domain of the CAR^23,24^.

In this study, we took a different approach to target TACI by generating an scFv sequence from TACI-immunized mice. While TACI has reported expression on activated T cells^37^, we did not detect staining on activated T cells using our G3D2 anti-TACI antibody. The only peripheral blood cells with minor staining were CD19^+^ cells, as would be expected. In addition, TACI has also been reported to be expressed on regulatory T cells^45^, making TACI especially appealing to target as a strategic approach to directly kill tumor as well as indirectly manipulate the immunosuppressive tumor microenvironment inflicted by regulatory T cells.

We demonstrated that T cells engineered to express a second-generation CAR based on an scFv directed against TACI had the ability to cure multiple myeloma tumors in xenograft models. The anti-TACI L-H orientation showed superior anti-tumor efficacy compared with its H-L counterpart. We observed that BCMA loss due to CRISPR/Cas9 deletion did not affect TACI expression on multiple myeloma cell line MM1S, confirming previous reports^23^. We also were able to validate the hypothesis that TACI surface expression is retained in patients who relapse with BCMA-negative disease after BCMA-targeted therapy with a primary patient sample. Even in cases with biallelic loss, as observed by Samur et al.^17^, TACI expression would be preserved as it is on another chromosome. Indeed, anti-TACI CAR T cells were able to induce regression of BCMA negative tumors in vivo and had a pro-inflammatory response to BCMA-negative patient multiple myeloma cells.

Loss of BCMA from anti-BCMA CAR T cell-treated patients likely reflects selective pressure on the antigen exerted by the CAR T cells^46^. Developing novel CARs against multiple surface antigens may prevent this problem. We therefore designed and validated the efficacy of a tandem CAR with scFvs directed against TACI and BCMA. Interestingly, we found that when target cells express both antigens, the anti-TACI-anti-BCMA configuration appeared slightly superior with regard to in vitro activity and in vivo tumor control. However, in the face of BCMA loss, this CAR configuration lost all activity, indicating its heavy dependence on BCMA expression. In contrast, in the face of BCMA loss induced by CRISPR/Cas9 or in a recurrent plasmacytoma after cilta-cel treatment in a patient, anti-BCMA-anti-TACI CARs retained activity; this leads us to conclude that this configuration is the superior dual-specific construct. Because of the widespread clinical use of BCMA-targeted therapies, we believe that there will be more selective pressure on BCMA, and therefore higher likelihood of loss of this antigen in patients with relapsed disease. In this setting, we would expect the anti-BCMA-anti-TACI CARs to have greater clinical impact based on their ability to target TACI. These data may suggest that for tandem bispecific scFvs, the scFv closer to the signaling domain has a stronger signal.

Because BCMA loss is relatively rare (4–33%) in patients treated with BCMA-targeting CARs^10,14,15^, one approach has been to target BCMA with higher-affinity or tighter-binding CARs, such as biparatopic binding domains. However, this approach may result in increased on-target toxicity, based on recent reports of Parkinsonian features reported in at least six anti-BCMA CAR T cell-treated patients^28–30^. These neurotoxicities occurred after a period of recovery from CRS. Symptoms included agitation, personality changes, short term memory loss, shuffling gait, difficulty swallowing, and joint stiffness, among others^30^. Further investigation discovered BCMA expression in the brain, on neurons and astrocytes in the basal ganglia^30^. In addition, BCMA RNA expression was validated in healthy brain tissue, suggesting that the resulting Parkinsonian features were due to on-target, off-tumor toxicity. Further investigation of three patients who died on trial showed no acute abnormalities via brain MRI. These patients experienced other adverse events, including sepsis and infection, but it is possible neurotoxicity contributed to the cause of death. It is possible that targeting a second antigen, such as TACI, could be a potentially safer alternative than increasing binding to BCMA avidity to BCMA, given our analysis showing limited expression of TACI in the basal ganglia. It should be noted that the dual targeting approach described would not prevent the BCMA toxicities and is limited in this regard, but the lack of overlapping toxicities is generally a feature sought in combination therapy strategies for cancer. Should these toxicities continue to be a main area of concern for BCMA therapy, targeting TACI alone would theoretically have a better safety profile than the anti-BCMA-anti-TACI approach.

In conclusion, we report potent anti-tumor activity of monospecific anti-TACI and bispecific anti-BCMA–anti-TACI CAR T cells in vitro and in xenograft models of multiple myeloma. These CAR T cells could offer an additional therapeutic option for patients with multiple myeloma or other plasma cell disorders.

## Methods

### Study design

This study was designed to develop anti-TACI directed CAR T cells for multiple myeloma. To validate the efficacy of these CARs, in vitro and in vivo functional assays were performed using a variety of multiple myeloma tumor cell lines. T cells were sourced from anonymized human blood samples. An Institutional Review Board protocol approved the purchase of anonymous human healthy donor leukapheresis products from the MGH blood bank. The Institutional Review Board (IRB) at the Massachusetts General Hospital determined the use of these T cells to be “non-human subjects research”. The Institutional Animal Care and Use Committee (IACUC) approved protocols for all animal work. Prior to CAR T cell treatment, animals were randomized. The multiple myeloma patient sample was obtained after written informed consent under protocol 16–206 that is approved by the Dana-Farber/Harvard Cancer Center IRB.

### RNA expression analysis

RNA microarray expression levels from the basal ganglia of 6 human donors were queried from the Allen Brain Atlas Data Portal. Dataset: Allen Institute for Brain Science (2010). Allen Human Brain Atlas: Microarray [TNFRSF13B; TNFRSF17]. Available from human.brain-map.org^35^. RRID:SCR_007416. Anatomic visualization was performed in Brain Explorer 2 (v2.3.5, Allen Institute for Brain Science)^47^. RNA single cell analysis expression data was assessed from the Human Protein Atlas^32^.

### Mice and cell lines

Male and female NOD-SCID-γ chain -/- (NSG) mice purchased from the Jackson laboratory were bred under pathogen-free conditions at the MGH Center for Cancer Research. Animals were maintained in rooms with 12:12 h light:dark cycles, humidity of 30–70%, and temperature of 21.1–24.5 C. All experiments were approved according to MGH Institutional Animal Care and Use Committee protocols. All cell lines (K562, MM1S, RPMI-8226, U266, SUPT1) were sourced from the American Type Culture Collection (ATCC). Cells were grown in conditions as recommended by ATCC. Tumor cells were transduced with enhanced GFP (eGFP) and click beetle green (CBG) luciferase. Cell lines were then sorted for 100% transduction on a BD FACSAria II or FACSAria Fusion cell sorter. For knockout generation, the MM1S cell line was transduced vectors obtained from the Broad Institute Genetic Perturbation Platform containing guide RNA and puromycin-resistance from the Brunello library (Brunello library) or electroporated with mRNA of the guide. Afterwards, cells were electroporated using a BTX ECM830 with CleanCap Cas9 mRNA (TriLink) and then again sorted for knockout by FACs. The following guides were used: BCMA TAACGCTGACATGTTAGAGG (BRDN0001494990) and TACI ACAATTCAGACTCGGGA (BRDN00016948784).

### TACI antibody development

Mice were immunized using commercially available TACI protein in collaboration with LifeTein LLC. LifeTein developed and screened hybridomas for anti-TACI positive antibodies. The resulting positive clone (G3D2) was sequenced (GenScript) and antibody isolated by LifeTein assessed for binding to TACI protein using surface plasma resonance (Affina Biotechnologies). Antibody was made 1 ug/mL in running buffer (PBS-P, 10 mM sodium phosphate, 150 nM NaCl, 0.005% Tween 20, pH 7.4) and captured on anti-mouse IgG surface (CM5 chip, 10,000RU anti-IgG antibody immobilized by standard amine-directed chemistry according to the manufacturer’s instructions; GE Healthcare Cat# BR-1008-38) to achieve 130 RU for TACI analysis. The flow rate was 50 uL/min with 2 min contact time and 5 min dissociation.

### Flow cytometry

Cells were washed with FACS buffer (2% FBS in PBS) and then incubated with antibody in the dark for 20 min at 4 C. Cells were then washed twice and DAPI was used to separate live/dead staining prior to analysis on a BD LSRFortessa X20 with the exception of Fig. 8g which was analyzed on a BD FACSLyric. Antibodies that required secondary staining had a similar protocol: after primary staining as described, the secondary was added before DAPI, again stained in the dark for 20 min at 4 C. The following antibodies were used: BCMA (19F2, BioLegend, 1:50 dilution), CCR7 (150503, BD Biosciences, 1:33 dilution), mouse CD11b (M1/70, Biolegend, 1:50 dilution), CD138 (MI15, BD Biosciences, 1:50 dilution), CD19 (SJ25C1, BD Biosciences, 1:33 dilution), CD27 (M-T271, BD Biosciences 1:50 dilution), CD27 Fig. 8g (L128, BD Biosciences, 1:50 dilution), CD3 (UCHT1, BD Biosciences, 1:50 dilution), CD38 (multi-epitope, Alpco Cytognos, 1:20 dilution), CD4 (SK3, BD Biosciences, 1:50 dilution), CD45 (HI30, BD Biosciences, 1:50 dilution), CD45RA (HI100, BD Biosciences, 1:100 dilution), CD56 (MY31, BD Biosciences, 1:50 dilution), CD56 Fig. 8g (HCD56, Biolegend, 1:25 dilution), CD69 (FN50, Biolegend, 1:100 dilution), CD8 (SK1, BD Biosciences, 1:50 dilution), CD95 (DX2, BD Biosciences, 1:50 dilution), cytoplasmic kappa (TB28-2, BD Biosciences, 1:10 dilution), cytoplasmic lambda (1-155-2, BD Biosciences, 1:10 dilution), HLA-DR (L243, BD Biosciences, 1:50 dilution), Lag-3 (T46-530, BD Biosciences, 1:50 dilution), mouse Ly-6G/Ly-6C (RB6-8C5, Biolegend, 1:50 dilution), mouse NK1.1 (PK136, Biolegend, 1:50 dilution), PD-1 (NAT105, Biolegend, 1:50 dilution), TACI (1A1, BioLegend; G3D2, Maus lab, 1.67ug/ml), anti-mouse IgG2b (RMG2b-1, BioLegend, 1:588 dilution), TACI Fig. 8h (1A1-K21-M22, BD Biosciences, 1:20 dilution), mouse TER-119 (TER-119, Biolegend, 1:50 dilution), TIM-3 (7D3, BD Biosciences, 1:50 dilution). For soluble protein binding assays the following were used: Human BCMA / TNFSRF17 Protein Fc Tag and Human TACI/TNFRSF13B Protein Fc Tag (ACROBiosystems) with the anti-human IgG Fc secondary (HP6017, BioLegend, 1:100 dilution). Protein was titrated and used to stain 50,000 CAR+ cells for 1 hr at 4 C. Cells were washed three times and stained for 20 min 4 C with secondary before being washed three times and analyzed by flow cytometry. For kappa/lambda staining of patient multiple myeloma cells, cells were stained for CD38, CD56, CD45, CD19, CD138, and CD27 for 15 min at 4 C, washed and resuspend in Cytofix/Cytoperm reagent (BD Biosciences) for 20 min at 4 C and then washed twice in Perm/Wash buffer (BD Biosciences) prior to kappa and lambda staining. Isotypes were used from the corresponding manufacturer throughout experiments. Trucount Absolute Counting Tubes (IVD BD Biosciences) were used according to manufacturer recommendations for CAR quantification in blood samples from in vivo. All data were collected using the BD FACSDiva software and analyzed using FlowJo Software.

### Construction of CARs

Monospecific and bispecific anti-TACI and BCMA (bb2121^48^) CAR T constructs were synthesized (GenScript) under the regulation of a human EF-1ɑ promoter and cloned into a third-generation lentiviral backbone. All CAR constructs contained a CD8 hinge and transmembrane domain, 4-1BB costimulatory domain, CD3ζ signaling domain, and fluorescent reporter mCherry to evaluate transduction efficiency.

### Lentiviral production

HEK293 T cells from ATCC were cultured in RPMI supplemented with 10% fetal bovine serum and 1% pencillin streptomycin. Synthesized CAR constructs were transfected with third generation packaging plasmids using Lipofectmine 3000 and P3000 (ThermoFisher Scientific) in OptiMEM (Gibco) media. Viral supernatants were harvested 24 and 48 h after transfection, combined, filtered and concentrated by ultracentrifugation (ThermoFisher Scientific Sorvall WX+ Ultracentrifuge) at 25000 RPM for 2 h at 4 C. Concentrated virus was stored at -80C and titered on SUPT1 cells to determine MOI for transduction of primary T cells via mCherry expression.

### CAR T cell production

An Institutional Review Board protocol approved purchase of anonymous human healthy donor leukapheresis products from the MGH blood bank. Stem Cell Technologies T cell Rosette Sep Isolation kit were used to isolate T cells. Bulk human T cells were activated on Day 0 using CD3/CD28 Dynabeads (Life Technologies) at a 1:3 T cell:bead ratio to generate CAR T cells and untransduced T cells from the same donors to serve as controls. T cells were grown in RPMI 1640 media with GlutaMAX and HEPES supplemented with 10% FBS, penicillin, streptomycin, and 20IU per ml recombinant human IL-2 (Peprotech). On Day 1 (24 h after activation) cells were transduced with CAR lentivirus at an MOI of 5. T cells continued expanding with media and IL-2 addition every 2–3 days. Dynabeads were removed via magnetic separation on Day 7 and cells were assessed by flow cytometry for mCherry expression on Days 12–14 to determine transduction efficiency. Prior to use in in vitro functional assays, CAR T cells and untransduced cells were thawed and rested for 2–24 h in IL-2.

### Cytotoxicity assays

CAR T cells were incubated with luciferase-expressing tumor cells at the indicated effector to target (E:T) cell ratios for 18 h. Cells were then lysed and luciferase activity was measured using a Synergy Neo2 luminescence microplate reader with Gen5 version 2.09 software (Biotek). The following formula was used to determine percentage lysis: (target cells alone relative luminescence units (RLU)–total RLU with CAR T cells)/(target cells alone RLU) x 100.

### Cytokine analysis

CAR T cells and tumor cells were cultured at a 1:1 ratio for 18 h with the exception of Fig. 8k which was a 2:1 E:T ratio for 24 h. Supernatants were then harvested and frozen for later cytokine analysis of cytokines. Cytokine expression was assessed with the Th1/Th2 Cytokine 11-Plex Human ProcartaPlex Panel (Invitrogen) according to the manufacturer’s instructions using a FLEXMAP 3D (Luminex, ThermoFisher Scientific).

### In vivo models

MM1S cells were administered intravenously with 1e6 cells in 100ul PBS and engrafted for 14 days prior to 5e6 (curative model) or 2e6 (stress model) CAR+ cells were administered intravenously in 100ul PBS. Animals were monitored biweekly for bioluminescent emission using an Ami HT optical imaging system (Spectral Instruments) following intraperitoneal substrate injection of D-Luciferin (30 mg/mL). All images were analyzed using Aura software. For the RPMI-8226 model, 5e6 tumor cells were administered subcutaneously in 200ul PBS and treated 14 days later with 2e6 CAR+ cells intravenously in 100ul PBS. Animals were monitored for tumor progression biweekly with caliper measurements with tumor volume calculated as length x width x (length + width)/2. Animals were euthanized as per the experimental protocol or when they met pre-specified endpoints defined by the IACUC. The maximal tumor size allowed was a diameter of 20 mm, which was not exceeded. One animal technician who performed all animal injections and monitoring was blinded to expected outcomes. All animal experiments included 5 animals per group. Mice were randomized post-tumor injection prior to treatment.

### Statistical methods

Analyses were performed with GraphPad Prism 9 (version 9.0). Unless otherwise stated, data were presented as mean +/– SEM and a 2-tailed Student t test. All tests were two-sided. Significance was considered for *P* < 0.05 as the following: *p* = *<0.05, **<0.01, ***<0.001, ****<0.0001.

### Reporting summary

Further information on research design is available in the Nature Portfolio Reporting Summary linked to this article.

### Supplementary information

Supplementary Information

Peer Review File

Reporting Summary

### Source data

Source Data

## Data Availability

Original data for graphs is provided in the Source Data file. For additional questions, please contact marcelavmaus@mgh.harvard.edu. The Allen Institute for Brain Science (2010) publicly available data used in this study are available in the Allen Brain Atlas Data Portal database under the Allen Human Brain Atlas: Microarray, Dataset: [TNFRSF13B; TNFRSF17]. Available from human.brain-map.org^35^. RRID:SCR_007416. The remaining data are available within the Article, Supplementary Information, or Source Data file. Source data are provided with this paper.
