# Supplementary material for: Anti-TACI single and dual-targeting CAR T cells overcome BCMA antigen loss in multiple myeloma
Source: Nat Commun. 2023 Nov 18;14:7509. doi: 10.1038/s41467-023-43416-7 (PMC10657357; doi:10.1038/s41467-023-43416-7)
Supplement: Supplementary file 1 — Supplementary Information [file 41467_2023_43416_MOESM1_ESM.pdf]

Supplementary Information

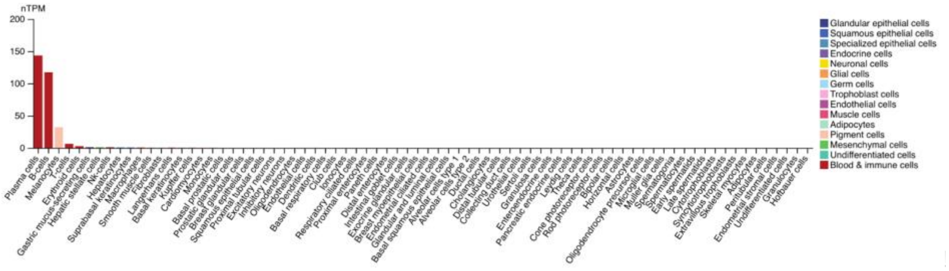

Supplementary Figure 1 TNFRSF13B RNA expression is enriched in plasma cells and B cells. Using the Human Protein Atlas, the *TNFRSF13B* gene was analyzed for single cell expression across cell types. Here cells are listed by descending expression, with enrichment shown for plasma cells and B cells. Image credit: Human Protein Atlas. Image available from: v21.1.proteinatlas.org.

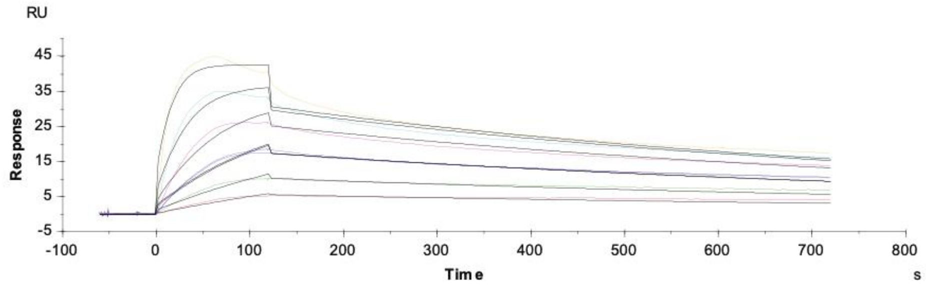

ka (1/Ms)	kd (1/s)	KD (M)	Rmax (RU)	Chi² (RU²)
1.407E+6	0.00121	8.61E-10	31.28	0.943

Supplementary Figure 2 G3D2 anti-TACI antibodies bind TACI protein. G3D2 antibody was captured using anti-mouse IgG immobilized by standard amine-directed chemistry. TACI protein binding is shown at 1.57, 3.13, 6.25 (2 replicates), 12.5, 25, and 50nM. Experiment was performed once. BCMA binding was also tested but very little binding was detected and its affinity could not be measured.

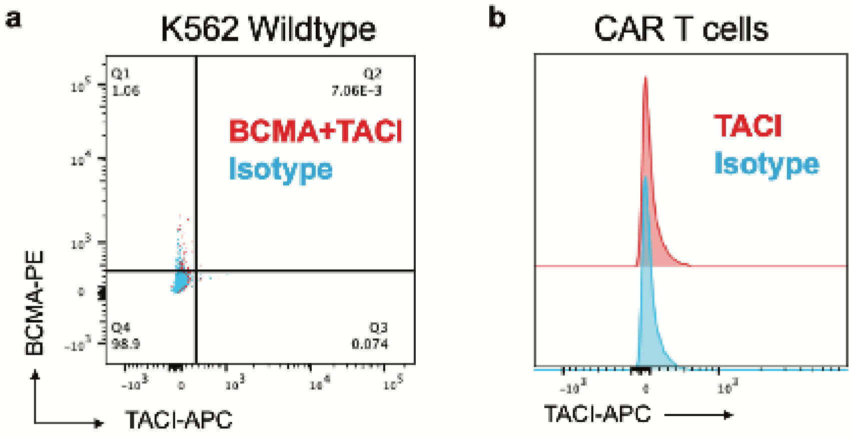

Supplementary Figure 3 Wildtype K562 and CAR T cells do not express TACI. a, BCMA and TACI staining of wildtype K562 cells using commercially available conjugated flow antibodies. **b**, TACI staining of CAR T cells against an irrelevant target (stained cells in red, isotype stain in blue).

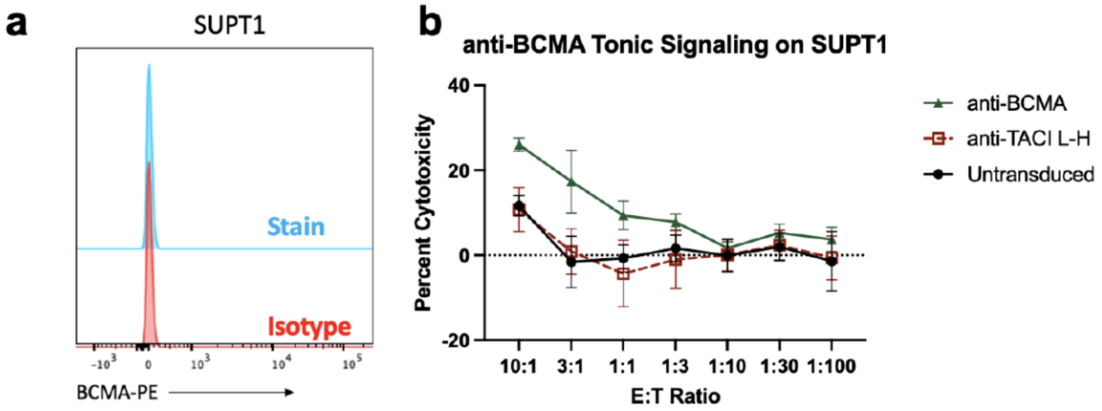

Supplementary Figure 4 BCMA CARs exhibit nonspecific killing against BCMA-negative SUPT1 cells. a, BCMA staining of SUPT1 lymphoma cells using a commercially available conjugated flow antibody. **b**, Luciferase-based cytotoxicity assay of SUPT1 cells targeted by CAR T cells at the indicated E:T ratios after 18 hours of coculture (biological triplicate). Calculated as a percentage of luminescence of tumor only wells. Raw data is provided in the Source Data file.

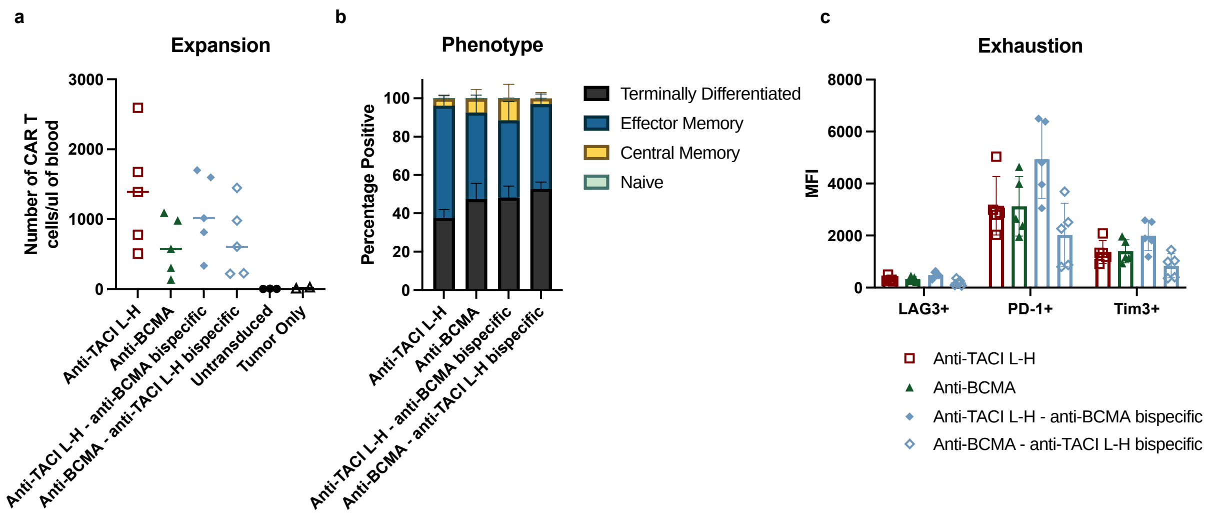

Supplementary Figure 5 Anti-TACI based CARs have similar phenotypes to anti-BCMA CARs in vivo. Blood from mice shown in Figure 7d-f were evaluated for CAR T cell expansion (**a**), phenotype (**b**) defined by CCR7 and CD45RA expression (terminally differentiated: CCR7-CD45RA+; effector memory: CCR7-CD45RA-; central memory: CCR7+CD45RA-; naïve: CCR7+CD45RA+) and exhaustion (**c**) at D28 post CAR treatment (n=5; ns for all). Raw data is provided in the Source Data file.

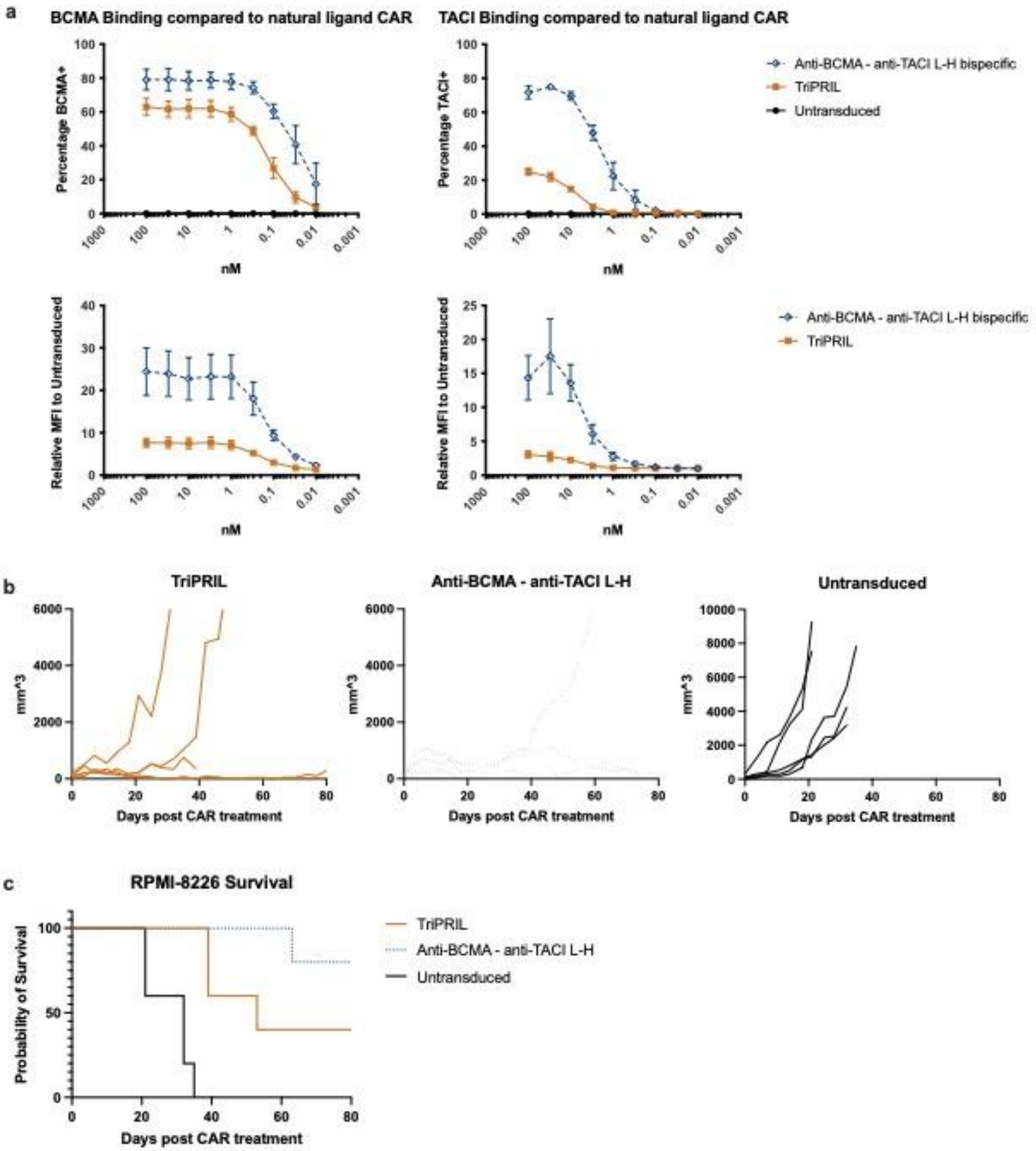

Supplementary Figure 6 Natural ligand-based CAR T cells targeting BCMA and TACI have less binding of soluble antigen and less efficacy in vivo compared to anti-BCMA-anti-TACI tandem bispecifics. A trimeric natural ligand based CAR termed TriPRIL was generated to compare to our tandem scFv design. **a**, Binding of soluble BCMA and TACI to TriPRIL and

anti-BCMA-anti-TACI L-H CARs at the indicated concentrations measured by flow cytometry. Percentage of TACI+ mcherry+ CAR T cells shown on top row and ratio of MFI of FITC on mCherry+ CAR T cells compared to Untransduced T cells in second row (n=3ND). Animals bearing RPMI-8226 subcutaneous tumors from Figure 7 d-f were also treated TriPRIL CARs and tumor growth (**b**) and survival were monitored (**c**) (5 mice per group). Raw data is provided in the Source Data file.
