# Supplementary material for: Anti-TACI single and dual-targeting CAR T cells overcome BCMA antigen loss in multiple myeloma
Source: Nat Commun. 2023 Nov 18;14:7509. doi: 10.1038/s41467-023-43416-7 (PMC10657357; doi:10.1038/s41467-023-43416-7)
Supplement: Supplementary file 3 — Reporting Summary [file 41467_2023_43416_MOESM3_ESM.pdf]

nature portfolio

Corresponding author(s): Marcela V. Maus

Last updated by author(s): Oct 29, 2023

Reporting Summary

Nature Portfolio wishes to improve the reproducibility of the work that we publish. This form provides structure for consistency and transparency in reporting. For further information on Nature Portfolio policies, see our Editorial Policies and the Editorial Policy Checklist.

Statistics

For all statistical analyses, confirm that the following items are present in the figure legend, table legend, main text, or Methods section.

n/a	Confirmed	
☐	☒	The exact sample size (*n*) for each experimental group/condition, given as a discrete number and unit of measurement
☐	☒	A statement on whether measurements were taken from distinct samples or whether the same sample was measured repeatedly
☐	☒	The statistical test(s) used AND whether they are one- or two-sided<br>*Only common tests should be described solely by name; describe more complex techniques in the Methods section.*
☒	☐	A description of all covariates tested
☐	☒	A description of any assumptions or corrections, such as tests of normality and adjustment for multiple comparisons
☐	☒	A full description of the statistical parameters including central tendency (e.g. means) or other basic estimates (e.g. regression coefficient) AND variation (e.g. standard deviation) or associated estimates of uncertainty (e.g. confidence intervals)
☐	☒	For null hypothesis testing, the test statistic (e.g. *F*, *t*, *r*) with confidence intervals, effect sizes, degrees of freedom and *P* value noted<br>*Give P values as exact values whenever suitable.*
☒	☐	For Bayesian analysis, information on the choice of priors and Markov chain Monte Carlo settings
☒	☐	For hierarchical and complex designs, identification of the appropriate level for tests and full reporting of outcomes
☒	☐	Estimates of effect sizes (e.g. Cohen's *d*, Pearson's *r*), indicating how they were calculated

Our web collection on statistics for biologists contains articles on many of the points above.

Software and code

Policy information about availability of computer code

Data collection	Flow cytometry was collected using BD FACSDiva software. Luciferase-based killing assays were measured using Gens version 2.09 Biotek software. In vivo imaging was collected using Aura software.
Data analysis	Flow cytometry was analyzed using FlowJo software. In vivo images were analyzed using Aura software. Validation analyses for in vitro and in vivo experiments utilized GraphPad Prism 9.0 software.

For manuscripts utilizing custom algorithms or software that are central to the research but not yet described in published literature, software must be made available to editors and reviewers. We strongly encourage code deposition in a community repository (e.g. GitHub). See the Nature Portfolio guidelines for submitting code & software for further information.

Data

Policy information about availability of data

All manuscripts must include a data availability statement. This statement should provide the following information, where applicable:

- Accession codes, unique identifiers, or web links for publicly available datasets
- A description of any restrictions on data availability
- For clinical datasets or third party data, please ensure that the statement adheres to our policy

Original data for graphs is provided in the Source Data file. For additional questions, please contact marcelavmaus@mgh.harvard.edu. The Allen Institute for Brain Science (2010) publicly available data used in this study are available in the Allen Brain Atlas Data Portal database under the Allen Human Brain Atlas: Microarray, Dataset: [TNFRSF13B:http://human.brain-map.org/microarray/search/show?exact_match=false&search_term="TNFRSF13B"&search_type=gene&page_num=0;

TNFRSF17: http://human.brain-map.org/microarray/search/show?exact_match=false&search_term="TNFRSF17"&search_type=gene&page_num=0]. Available from human.brain-map.org35. RRID:SCR_007416. The remaining data are available within the Article, Supplementary Information, or Source Data file.

Research involving human participants, their data, or biological material

Policy information about studies with human participants or human data. See also policy information about sex, gender (identity/presentation), and sexual orientation and race, ethnicity and racism.

Reporting on sex and gender	One patient sample was used for this study. It came from a 56-year-old male with relapsed myeloma.
Reporting on race, ethnicity, or other socially relevant groupings	Only one patient sample was used in this study, so we did not control for race or ethnicity as a confounding variable.
Population characteristics	See above.
Recruitment	The patient sample was identified as BCMA-negative from a cohort of patients treated with commercially available CART cell products under DF/HCC protocol 16-206.
Ethics oversight	The patient sample was obtained after written informed consent under IRB-approved protocol 16-206 at Dana-Farber/ Harvard Cancer Center (DF/HCC).

Note that full information on the approval of the study protocol must also be provided in the manuscript.

Field-specific reporting

Please select the one below that is the best fit for your research. If you are not sure, read the appropriate sections before making your selection.

☒ Life sciences ☐ Behavioural & social sciences ☐ Ecological, evolutionary & environmental sciences

For a reference copy of the document with all sections, see nature.com/documents/nr-reporting-summary-flat.pdf

Life sciences study design

All studies must disclose on these points even when the disclosure is negative.

Sample size	No statistical methods were used to pre-determine sample sizes, but all major experiments were performed with at least three biological replicates to ensure findings were reproducible, with the exception of the patient sample testing where the one BCMA-negative sample was tested in technical duplicate due to limited sample material. Based on prior experience, we estimated that statistically meaningful differences between groups of mice would require at least 4 animals.
Data exclusions	No data were excluded from analysis.
Replication	All major experiments were run with at least three biological replicates with the exception of the patient sample testing where the one BCMA-negative sample was tested in technical duplicate due to limited sample material. Technical replicates for major findings are noted in the figure legends. Attempts at experimental replication were successful.
Randomization	Mice were randomized prior to CAR-T treatment to ensure equivalent tumor burden among groups. For in vitro experiments, groups were randomly assigned.
Blinding	One veterinary technician was in charge of injecting all mice for bioluminescent readings and was blinded to the expected outcomes. In vitro experiments were not blinded due to the non-biased collection of data using computer software.

Reporting for specific materials, systems and methods

We require information from authors about some types of materials, experimental systems and methods used in many studies. Here, indicate whether each material, system or method listed is relevant to your study. If you are not sure if a list item applies to your research, read the appropriate section before selecting a response.

Materials & experimental systems

n/a	Involved in the study	
	x	Antibodies
	x	Eukaryotic cell lines
x		Palaeontology and archaeology
	x	Animals and other organisms
x		Clinical data
x		Dual use research of concern
x		Plants

Methods

n/a	Involved in the study	
x		ChIP-seq
	x	Flow cytometry
x		MRI-based neuroimaging

Antibodies

Antibodies used	The following antibodies were used: BCMA (19F2, Biolegend), CCR7 (150503, BD Biosciences), mouse CDllb (Ml/70, Biolegend), CD138 (Ml15, BD Biosciences), CD19 (SJ25Cl, BD Biosciences), CD27 (M-T271, BD Biosciences), CD27 Figure 8g (L128, BD Biosciences), CD3 (UCHTl, BD Biosciences), CD38 (multi-epitope, Alpco Cytognos), CD4 (SK3, BD Biosciences), CD45 (Hl30, BD Biosciences), CD45RA (HllO0, BD Biosciences), CD56 (MY31, BD Biosciences), CD56 Figure 8g (HCD56, Biolegend), CD69 (FN50, Biolegend), CDS (SKl, BD Biosciences), CD95 (DX2, BD Biosciences), cytoplasmic kappa (TB28-2, BD Biosciences), cytoplasmic lambda (1-155-2, BD Biosciences), HLA-DR (L243, BD Biosciences), Lag-3 (T46-530, BD Biosciences), mouse Ly-6G/Ly-6C (RB6-8C5, Biolegend), mouse NKl.1 (PK136, Biolegend), PD-1 (NAT105, Biolegend), TACI (lAl, Biolegend; G3D2, Maus lab), anti-mouse lgG2b (RMG2b-1, Biolegend); TACI Figure 8h (1Al-K21-M22, BD Biosciences), mouse TER-119 (TER-119, Biolegend), TIM-3 (7D3, BD Biosciences).
Validation	Each primary antibody has been validated by the manufacturer for use to detect human species targets. We validated our novel TACI antibody on overexpressing cell lines.

Eukaryotic cell lines

Policy information about cell lines and Sex and Gender in Research

Cell line source(s)	All cell lines (K562, MMlS, RPMl-8226, U266, SUPTl) were sourced from the American Type Culture Collection (ATCC).
Authentication	Cell lines were authenticated using STR profiling at least once every 3 years from receipt.
Mycoplasma contamination	All cell lines tested negative for mycoplasma contamination.
Commonly misidentified lines (See ICLAC register)	No commonly misidentified cell lines were used in the study.

Animals and other research organisms

Policy information about studies involving animals; ARRIVE guidelines recommended for reporting animal research, and Sex and Gender in Research

Laboratory animals	Male and female NSG mice aged 6-12 weeks were used for experiments.
Wild animals	The study did not involve wild animals.
Reporting on sex	Both male and female mice were used in animal experiments.
Field-collected samples	The study did not involve field-collected samples.
Ethics oversight	Experiments were performed according to MGH Institutional Animal Care and Use Committee approved protocols.

Note that full information on the approval of the study protocol must also be provided in the manuscript.

Plants

Seed stocks

Report on the source of all seed stocks or other plant material used. If applicable, state the seed stock centre and catalogue number. If plant specimens were collected from the field, describe the collection location, date and sampling procedures.

Novel plant genotypes

Describe the methods by which all novel plant genotypes were produced. This includes those generated by transgenic approaches, gene editing, chemical/radiation-based mutagenesis and hybridization. For transgenic lines, describe the transformation method, the number of independent lines analyzed and the generation upon which experiments were performed. For gene-edited lines, describe the editor used, the endogenous sequence targeted for editing, the targeting guide RNA sequence (if applicable) and how the editor was applied.

Authentication

Describe any authentication procedures for each seed stock used or novel genotype generated. Describe any experiments used to assess the effect of a mutation and, where applicable, how potential secondary effects (e.g. second site T-DNA insertions, mosiacism, off-target gene editing) were examined.

Flow Cytometry

Plots

Confirm that:

☒ The axis labels state the marker and fluorochrome used (e.g. CD4-FITC).

☒ The axis scales are clearly visible. Include numbers along axes only for bottom left plot of group (a 'group' is an analysis of identical markers).

☒ All plots are contour plots with outliers or pseudocolor plots.

☒ A numerical value for number of cells or percentage (with statistics) is provided.

Methodology

Sample preparation

Tumor cells were cultured as recommended by ATCC, harvested, and lentivirally transduced or electroporated to create gene KO or overexpression cell lines. CART cells were generated from healthy donors with lentiviral transduction.

Instrument

BD Fortessa-X20

Software

FlowJo

Cell population abundance

Post-sort purity was always assessed and confirmed prior to use in experiments.

Gating strategy

Typically cells were assessed with DAPI viability and gated on single cells. Tumor cells were differentiated as GFP. Positive and negative gates were determined based on isotype or WT controls.

☒ Tick this box to confirm that a figure exemplifying the gating strategy is provided in the Supplementary Information.
